# Glycyrrhizic Acid and Compound Probiotics Supplementation Alters the Intestinal Transcriptome and Microbiome of Weaned Piglets Exposed to Deoxynivalenol

**DOI:** 10.3390/toxins14120856

**Published:** 2022-12-04

**Authors:** Xiaoxiang Xu, Juan Chang, Ping Wang, Chaoqi Liu, Mengjie Liu, Ting Zhou, Qingqiang Yin, Guorong Yan

**Affiliations:** 1 Shanghai Skin Disease Hospital, School of Medicine, Tongji University, Shanghai 200443, China; 2 College of Animal Science and Technology, Henan Agricultural University, Zhengzhou 450046, China; 3 Guelph Research and Development Centre, Agriculture and Agri-Food Canada, Guelph, ON N1G 5C9, Canada

**Keywords:** deoxynivalenol, glycyrrhizic acid, compound probiotics, transcriptome, gut microbiota, piglets

## Abstract

Deoxynivalenol (DON) is a widespread mycotoxin that affects the intestinal health of animals and humans. In the present study, we performed RNA-sequencing and 16S rRNA sequencing in piglets after DON and glycyrrhizic acid and compound probiotics (GAP) supplementation to determine the changes in intestinal transcriptome and microbiota. Transcriptome results indicated that DON exposure altered intestinal gene expression involved in nutrient transport and metabolism. Genes related to lipid metabolism, such as PLIN1, PLIN4, ADIPOQ, and FABP4 in the intestine, were significantly decreased by DON exposure, while their expressions were significantly increased after GAP supplementation. KEGG enrichment analysis showed that GAP supplementation promoted intestinal digestion and absorption of proteins, fats, vitamins, and other nutrients. Results of gut microbiota composition showed that GAP supplementation significantly improved the diversity of gut microbiota. DON exposure significantly increased *Proteobacteria*, *Actinobacteria*, and *Bacillus* abundances and decreased *Firmicutes*, *Lactobacillus*, and *Streptococcus* abundances; however, dietary supplementation with GAP observably recovered their abundances to normal. In addition, predictive functions by PICRUSt analysis showed that DON exposure decreased lipid metabolism, whereas GAP supplementation increased immune system. This result demonstrated that dietary exposure to DON altered the intestinal gene expressions related to nutrient metabolism and induced disturbances of intestinal microbiota, while supplementing GAP to DON-contaminated diets could improve intestinal health for piglets.

## 1. Introduction

Deoxynivalenol (DON), produced by *Fusarium* spp., belongs to the trichothecenes toxin, which is one of the most common mycotoxins usually detected in crops, feedstuffs, and animal diets worldwide. A recent research reported that the concentration of DON exceeded the permissible levels in most samples of food and feeds [1]. In China, the DON containment is more serious and was found in all the wheat samples and 99.8% maize samples [2]. Ingestion of DON can induce adverse health to animals and humans. On the other hand, DON can cause acute and chronic toxicity such as cytotoxicity, immunotoxicity and reproductive toxicity, and even carcinogenicity [3]. It was reported that swine is more sensitive to DON than the other kinds of animals [4]. Therefore, studying the toxicity mechanism of DON for swine is of great significance in evaluating the ecological safety of DON pollution. Moreover, alleviative strategies regarding the DON-induced toxicity also need to be conducted to reduce DON toxicity in pig industry.

Due to DON’s toxic properties and high stability, its presence in the food chain increases the risks to human health and animal productivity [5]. Some methods including physical, chemical, biological, irradiation, ultrasound, and ozone treatments have been reported to decrease DON toxicity [6]. The efficacy and mechanism of different methods varied considerably, leading to various degradation byproducts with different toxicity. Among these methods, one biological approach for the detoxification of DON depends on the adsorption characteristics of glucomannan from the cell wall of microorganisms [7]. The strong binding capacity between the adsorbent such as lactic acid bacteria and the mycotoxin can compete for the adsorption of the mycotoxin by the digestive tract, leading to a physical removal of DON [8]. Many studies have proven the detoxification capacity of probiotic bacteria such as lactic acid bacteria [9], *Saccharomyces cerevisiae* (*S. cerevisiae*) [10], and *Enterococcus faecalis* (*E. faecalis*) [11], which could improve gastrointestinal function and animal production performance and regulate the composition of intestinal microflora. In addition, glycyrrhizin (GA) is an extract from glycyrrhiza, which can promote the growth and meat quality in piglets and alleviate DON-induced oxidative stress, inflammatory response, and apoptosis [12]. Moreover, our primary study has shown that the combination of compound probiotics with berberine could enhance host intestinal homeostasis by modulating the composition of gut microbiota for improving piglet production performance [13]. On the other hand, our previous study also reported that *S. cerevisiae* and *E. faecalis* combined with glycyrrhizic acid (GA) have the ability to alleviate the DON-induced cell damage in vitro [14]. Meanwhile, dietary glycyrrhizic acid and compound probiotics (GAP) supplementation in a DON-contaminated diet could significantly promote growth performance and alleviate DON-induced intestinal damage of weaned piglets. Based on these findings, we want to further demonstrate the differences in intestinal transcriptome and microbiome of weaned piglets exposed to DON by dietary GAP supplementation.

In recent years, with the rapid development of next-generation sequencing, RNA-sequencing (RNA-seq) has become one of the important means to study gene function and screen new genes and has been used extensively in livestock breeding and disease diagnosis. It was reported that through transcriptional analysis of pig brain tissue for the first time, potential genes and biological pathways related to feed conversion efficiency in pigs were found, indicating that transcription factors NR2F2, TFAP2D, and HNF1B affect feed conversion rate mainly by regulating feeding behavior, insulin sensitivity, and energy metabolism [15]. In addition, 16S rRNA gene sequencing technology has been widely used to analyze the structure of intestinal microbiota. Guo et al. studied the relationship between ochratoxin A (OTA) in food and intestinal microbes by 16S rRNA gene sequencing technology and found that OTA could significantly reduce the diversity of intestinal microbiota and change its function and found that *Lactobacillus* played a key role in the process of OTA detoxification [16]. Hence, the present study aimed to reveal the mechanism of GAP supplementation on alleviating DON-induced damages by using intestinal transcriptome and microbiome analyses in weaned piglets.

## 2. Results

### 2.1. Transcriptional Changes of Piglets with GAP Supplementation

**Figure 1:**
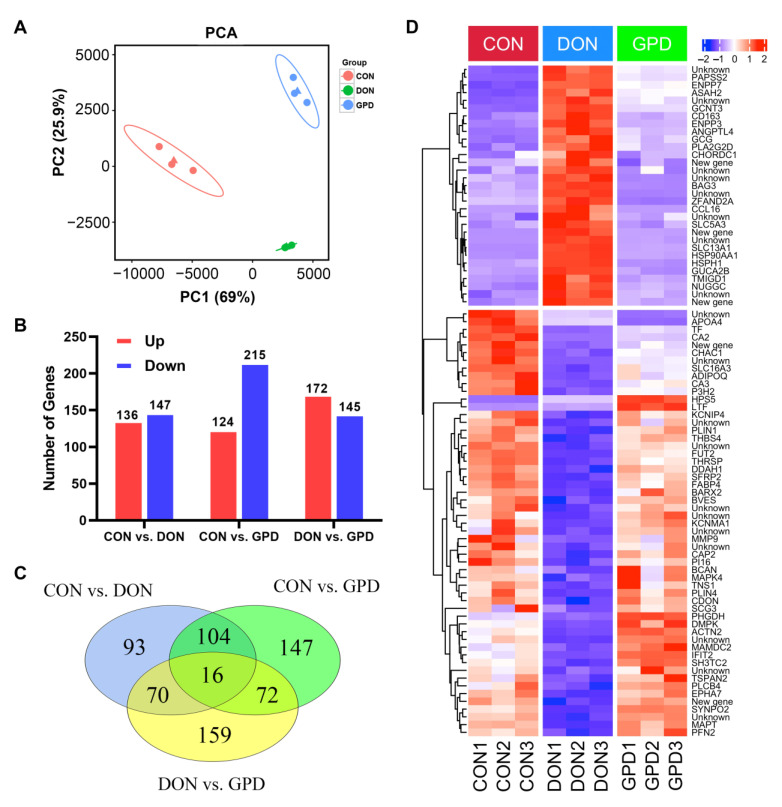
Identification of differentially expressed genes in the jejunum. (**A**) Principal component analyses of relationships among nine samples and the triangles in red, green and blue represents the mean value of all samples in the CON, DON and GPD, respectively. (**B**) Differentially expressed genes between CON and DON groups, CON and GPD groups, and DON and GPD groups, respectively. Red and blue colors represent up-regulated and down-regulated genes, respectively. (**C**) Venn diagram for the DEGs. (**D**) Heatmap for 86 DEGs co-expressed in CON vs. DON and DON vs. GPD groups. CON, normal basal diet; DON, DON-contaminated diet containing 1040 µg/kg DON; GPD (GAP + DON), DON-contaminated diet containing 1040 µg/kg DON supplemented with 400 mg/kg GA, 1 × 10^6^ CFU/g *S. cerevisiae*, and 1 × 10^6^ CFU/g *E. faecalis*.

We first investigated the jejunum transcriptional alterations associated with DON challenge with or without GAP in piglets using RNA-seq. The control group (CON): the piglets fed with a basal diet; the DON-contaminated group (DON): the piglets fed with a DON-contaminated diet; the GAP+DON group (GPD): the piglets fed with a DON-contaminated diet supplemented with GAP. The principal component analysis (PCA) result is shown in Figure 1A. All samples from the different groups were gathered into an individual group, indicating a distinct gene expression mode in these three groups. About 283, 339, and 317 differentially expressed genes (DEGs) (*p*-value < 0.05 and |log_2_ fold change| > 1) were identified in CON vs. DON, CON vs. GPD, and DON vs. GPD, respectively (Figure 1B). Venn diagram analysis indicated that 86 DEGs were co-expressed in CON vs. DON and DON vs. GPD, and a total of 16 DEGs were co-expressed in CON vs. DON, CON vs. GPD, and DON vs. GPD (Figure 1C). The expressions of these 86 DEGs were also presented in the heatmap (Figure 1D). In addition, we also found that among the 86 co-expressed DEGs, 31 DEGs, including heat-shock protein 90 alpha family class A member 1 (HSP90AA1), guanylate cyclase activator 2B (GUCA2B), angiopoietin-like protein 4 (ANGPTL4), solute carrier family 5 member 3 (SLC5A3), and other genes, were significantly up-regulated in CON vs. DON but down-regulated in DON vs. GPD; and 51 DEGs such as perilipin 1 (PLIN1), perilipin 4 (PLIN4), phosphoglycerate dehydrogenase (PHGDH), matrix metallopeptidase 9 (MMP9), fucosyltransferase 2 (FUT2), actinin alpha 2 (ACTN2), adiponectin C1Q and collagen domain containing (ADIPOQ), fatty acid binding protein 4 (FABP4), and other genes were significantly down-regulated in CON vs. DON group but up-regulated in DON vs. GPD group (Table S1).

### 2.2. QRT-PCR Validation

**Figure 2:**
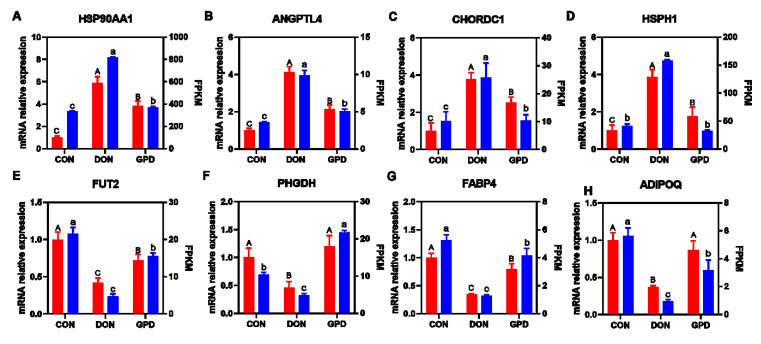
The qPCR validation of the RNA-seq. (**A**–**H**) The relative mRNA expressions of qRT-PCR and FPKM of RNA-seq analysis of the HSP90AA1, ANGPTL4, CHORDC1, HSPH1, FUT2, PHGDH, FABP4, and ADIPOQ genes, respectively. The red represents the mRNA relative expressions of qRT-PCR, and the blue represents FPKM value of RNA-seq. All values are expressed as the means ± SD (n = 3). Values with different capital or lowercase letter indicate a significant difference (*p* < 0.05), while those with the same letter indicate no significant difference (*p* > 0.05).

To validate the gene expression profile from RNA-seq, eight genes (HSP90AA1, ANGPTL4, cysteine- and histidine-rich domain-containing 1 (CHORDC1), heat-shock 105kDa/110kDa protein 1 (HSPH1), FUT2, PHGDH, FABP4, and ADIPOQ) were selected to validate by the qRT-PCR assay. Relative expression levels from qRT-PCR and FPKM values from the transcriptomic data are shown in Figure 2A–H. The results showed that the expression trend of these genes validated by qRT-PCR was in accordance with the results from RNA-seq analysis.

### 2.3. Functional Annotation Analyses

**Figure 3:**
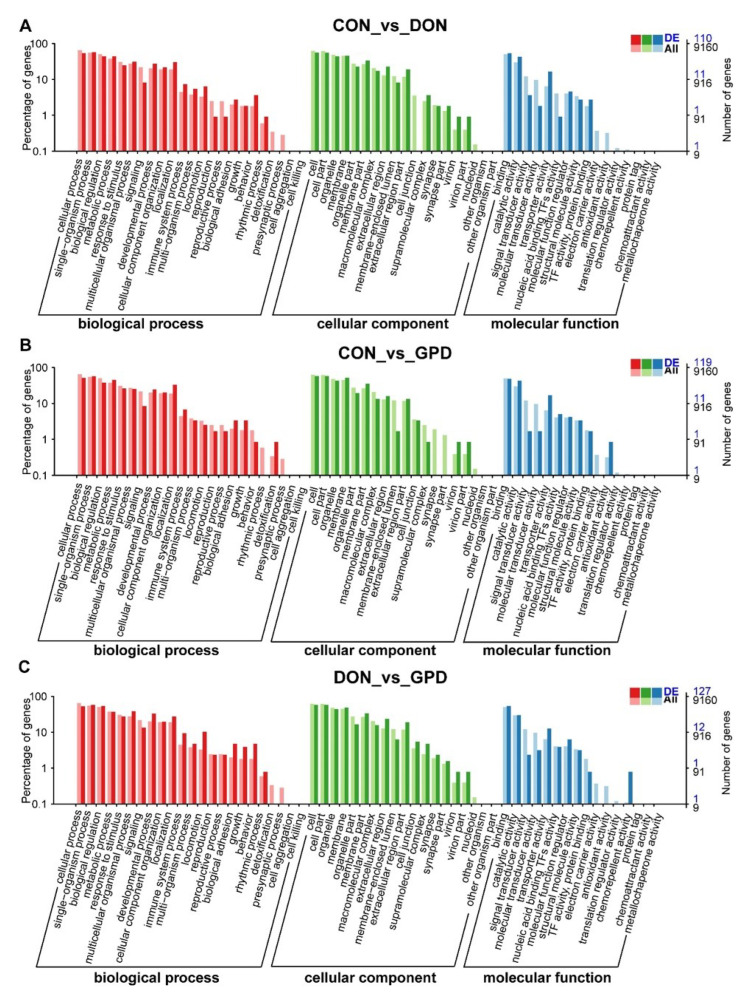
GO enrichment analysis of differentially expressed genes. (**A**–**C**) GO enrichment analysis of differentially expressed genes among CON vs. DON, CON vs. GPD, and DON vs. GPD groups.

To explore the biological processes and pathways of DEGs, the DEGs were functionally annotated using the *Gene Ontology* (GO) and *Kyoto Encyclopedia of Genes and Genomes* (KEGG) databases. They were significantly enriched in 58 GO terms in three categories including biological process, molecular function, and cellular component. As shown in Figure 3A–C, the GO enrichment analysis indicated that the DEGs in different treatment groups had similar enrichment categories, which were mainly involved in cellular process, metabolic process, stimulus response, cells, organelles, binding, and catalytic activity. Furthermore, the most significant difference was observed in the level of biological process, mainly including cellular process, metabolic process, biological regulation, and response to stimulus, etc., indicating that DON may seriously affect the biological processes in the jejunum of piglets, leading to cell cycle arrest and apoptosis.

**Figure 4:**
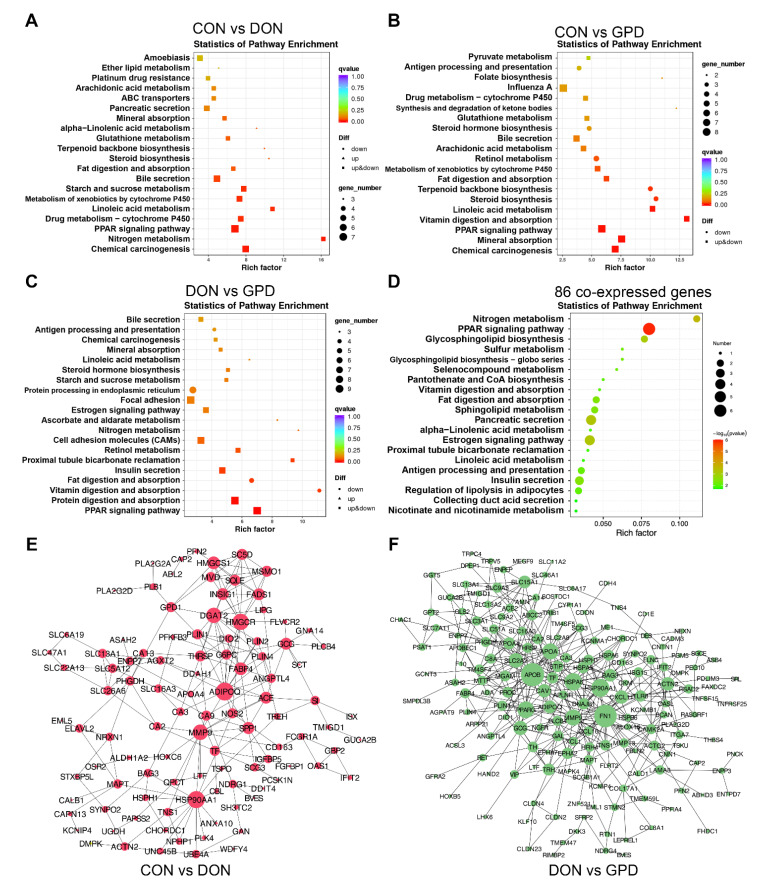
KEGG and PPI analysis of differential expressed genes. (**A**–**C**) KEGG enrichment analysis of differentially expressed genes in CON vs. DON group, CON vs. GPD group, and DON vs. GPD group. (**D**) KEGG enrichment analysis of 86 co-expressed differential genes in CON vs. DON and DON vs. GPD groups. (**E**) PPI analysis of differential expressed genes in CON vs. DON group; (**F**) PPI analysis of differential expressed genes in DON vs. GPD group.

The following KEGG pathway enrichment analysis was also performed using the DEGs from different comparisons. In CON vs. DON group (Figure 4A), the top 10 pathways were chemical carcinogenesis, nitrogen metabolism, PPAR signaling pathway, drug metabolism-cytochrome P450, linoleic acid metabolism, metabolism of xenobiotics by cytochrome P450, starch and sucrose metabolism, bile secretion, fat digestion and absorption, and steroid biosynthesis. It was found that some down-regulated DEGs were mainly enriched in steroid biosynthesis and terpenoid backbone biosynthesis. In CON vs. GPD group (Figure 4B), the top 10 pathways were chemical carcinogenesis, mineral absorption, PPAR signaling pathway, vitamin digestion and absorption, linoleic acid metabolism, steroid biosynthesis, terpenoid backbone biosynthesis, fat digestion and absorption, metabolism of xenobiotics by cytochrome P450, and retinol metabolism. In DON vs. GPD group (Figure 4C), the top 10 pathways were PPAR signaling pathway, protein digestion and absorption, vitamin digestion and absorption, fat digestion and absorption, insulin secretion, proximal tubule bicarbonate reclamation, retinol metabolism, cell adhesion molecules (CAMs), nitrogen metabolism, and ascorbate and aldarate metabolism. It was found that the up-regulated DEGs in GPD group were enriched in nitrogen metabolism, and the down-regulated DEGs were enriched in vitamin digestion and fat digestion and absorption. In addition, the KEGG enrichment among the 86 co-expressed DEGs in CON vs. DON and DON vs. GPD were investigated, which were mainly involved in PPAR signaling pathway, nitrogen metabolism, protein digestion and absorption, linoleic acid metabolism, estrogen signaling pathway, alpha-linoleic acid metabolism, and fat digestion and absorption (Figure 4D), suggesting that GAP addition promoted the digestion and absorption of nutrients such as protein, fat, and vitamins in the intestine.

To further investigate the interactions among the identified DEGs, protein–protein interaction (PPI) analyses were performed in the CON vs. DON and DON vs. GPD groups. As shown in Figure 4E,F, both groups built an independent network, respectively. The CON vs. DON group mainly included ADIPOQ, HSP90AA1, MMP9, FABP4, and other related genes, among which ADIPOQ was the hub gene with the most interaction with other genes. The network in DON vs. GPD group mainly included apolipoprotein B (APOB), fibronectin 1 (FN1), HSP90AA1, peroxisome proliferative activated receptor gamma (PPARG), apolipoprotein A 1 (APOA1), MMP9, solute carrier family 2 member 2 (SLC2A2), ACTN2, and apolipoprotein A 4 (APOA4)-related genes, and APOB was the hub gene with the most interaction with other genes, followed by FN1.

### 2.4. Differences of Microbiota Composition in Jejunum Contents

**Figure 5:**
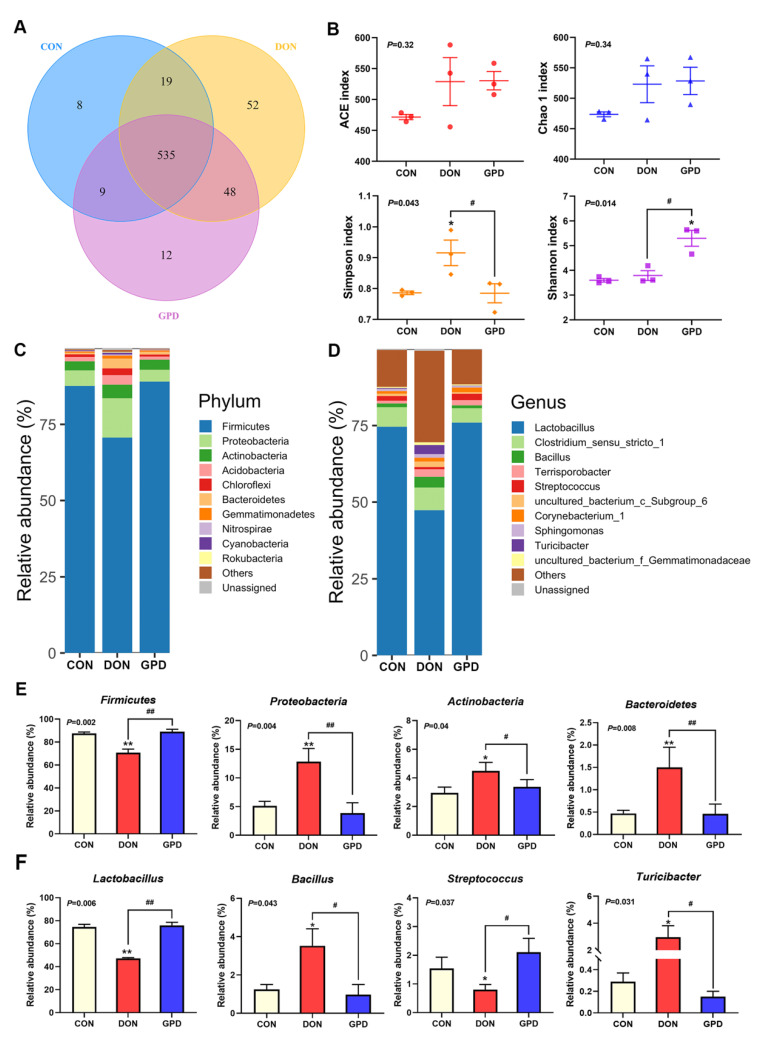
Effects of GAP on microbiota in jejunum contents of piglets challenged with DON. (**A**) Venn diagram was generated to compare operational taxonomic units (OTUs) among different groups. (**B**) The diversity and richness indexes of microbiota in each group and each point in the figure represents the index of one sample. (**C**,**D**) Microbiota compositions at the phylum and genus levels, respectively. (**E**,**F**) The relative abundances of some significant-alteration microbiota at the phylum and genus levels among three groups, respectively. Compared with the control group, * *p* < 0.05, ** *p* < 0.01; compared with the DON group, ^#^ *p* < 0.05, ^##^ *p* < 0.01.

In order to investigate the effect of DON exposure and GAP supplementation on gut bacterial composition, the jejunum contents of piglets from three groups were compared using 16S rRNA sequencing. A total of 491,857 high-quality sequence reads were generated with an average of 54,651 reads per sample after filtering out low-quality sequences. At 97% identity level, 571, 654, and 604 operational taxonomic units (OTUs) were detected in the fecal samples of piglets from CON, DON, and GPD groups, respectively. Interestingly, more OTUs were identified in DON-treated groups. Overall, 535 common OTUs and 72 unique OTUs were isolated in these three groups (Figure 5A). Simpson index in CON and GPD groups was significantly lower than that in DON group (*p* < 0.05), and the Shannon index in GPD group was significantly higher than that in CON and DON groups (*p* < 0.05), which indicated that dietary GAP increased the microbial diversity. However, there were no significant differences in the richness estimators (Chao and ACE index) among three groups (*p* > 0.05) (Figure 5B). The gut microbiota of piglets exhibited individual variation, while *Firmicutes* (82.45%) and *Proteobacteria* (7.27%) were the two main dominant phyla (Figure 5C). Meanwhile, at the genus level, *Lactobacillus* (65.96%), *Clostridium_sensu_stricto_1* (6.14%), *Bacillus* (1.91%), *Terrisporobacter* (1.66%), *Streptococcus* (1.48%), *Corynebacterium_1* (1.24%), *Turicibacter* (1.14%), *uncultured_bacterium_c_Subgroup_6* (0.99%), *Sphingomonas* (0.77%), and *uncultured_bacterium_f_Gemmatimonadaceae* (0.6%) were top 10 genera (Figure 5D). Moreover, significant differences in the relative abundances of gut microbiota among the three groups were further identified (Figure 5E,F). The relative abundance of *Proteobacteria* (*p* < 0.01), *Actinobacteria* (*p* < 0.01), *Bacteroidetes* (*p* < 0.01), *Bacillus* (*p* < 0.05), and *Turicibacter* (*p* < 0.01) in the piglets challenged with DON were significantly increased compared with CON group; however, they were significantly decreased by dietary supplementation with GAP (*p* < 0.05). The piglets challenged with DON significantly decreased in abundances of *Firmicutes* (*p* < 0.01), *Lactobacillus* (*p* < 0.01), and *Streptococcus* (*p* < 0.05) compared with CON group, while GAP supplementation increased their relative abundances (*p* < 0.05).

### 2.5. LEfSe and Predictive Function Analysis

**Figure 6:**
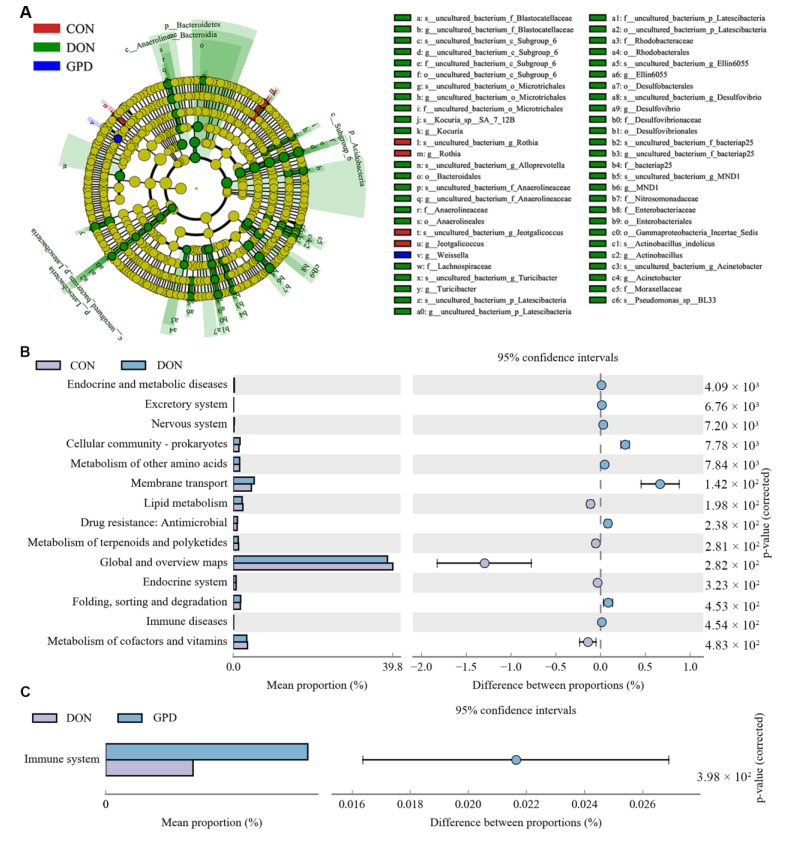
Linear discriminant analysis effect size (LefSe) and KEGG function prediction of the piglet intestinal microbiota. (**A**) Cladogram of the microbial communities in intestinal microbiota. (**B**) Prediction of functional differences of related microbiota between CON and DON groups. (**C**) Prediction of functional differences of related microbiota between DON and GPD groups.

Linear discriminant analysis effect size (LefSe) revealed that the DON group in the jejunum had more differential biomarkers (Figure 6A). Subsequently, the potential function of gut microbiota in piglets was analyzed by the PICRUSt program. Compared with the CON group, metabolic pathways including lipid metabolism, cofactors, and vitamin metabolism in DON group were significantly down-regulated, whereas nervous system, membrane transport, and immune diseases were significantly up-regulated (Figure 6B). Furthermore, the immune system in the GPD group was significantly elevated compared to the DON group (Figure 6C), which indicated that GAP supplementation could improve the immunity of piglets challenged with DON.

### 2.6. Correlation Analysis between the DEGs and Gut Microbiota

**Figure 7:**
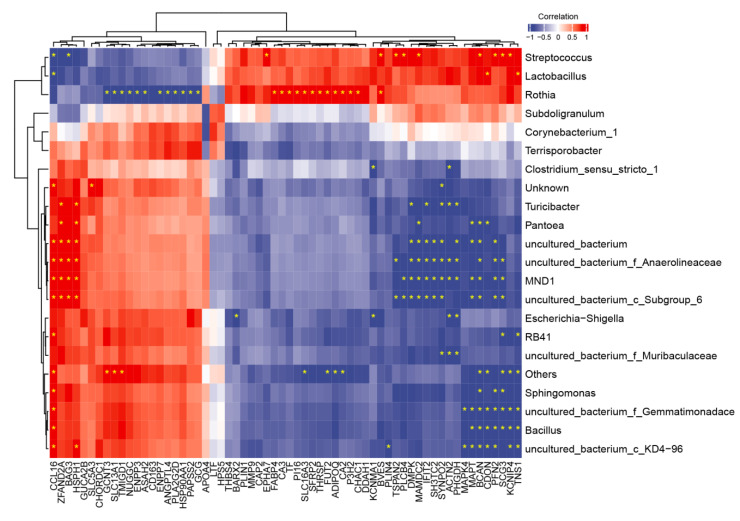
Correlation clustering heatmap analysis between co-expressed differential genes and microbiota in piglet jejunum contents at genus level; * indicates *p* < 0.05; red represents a positive correlation, blue represents a negative correlation, and white represents no correlation.

In order to explore the interaction between differentially expressed genes and intestinal microflora, Spearman correlation analysis based on 62 known co-expressed DEGs in jejunum tissues and the top 20 genera in jejunum contents was conducted. The results are shown in Figure 7. The correlation between intestinal microbiota and co-expressed differentially expressed genes was obviously divided into a “field pattern”. *Streptococcus*, *Lactobacillus*, and *Rothia* were positively correlated with tensin 1 (TNS1), Kv channel interacting protein 4 (KCNIP4), secretogranin 3 (SCG3), brevican (BCAN), tetraspanin 2 (TSPAN2), and SLC16A3 genes. Most of these genes were down-regulated in CON vs. DON group and up-regulated in DON vs. GPD group. They were negatively correlated with C-C motif chemokine 16 (CCL16), Bcl 2-associated athanogene 3 (BAG3), solute carrier family 13 member 1 (SLC13A1), ANGPTL4, and glucagon (GCG) genes, and most of these genes were up-regulated in CON vs. DON group and down-regulated in DON vs. GPD group. *Lactobacillus* was positively correlated with TNS1 and cell-adhesion-associated oncogene regulated (CDON) and negatively correlated with CCL16. *Streptococcus* was negatively correlated with CCL6 and BAG3 and positively correlated with KCNIP4, SCG3, profilin 2 (PFN2), and BCAN genes. However, the results of other genera were opposite to those of the above three genera. Among them, *Escherichia-Shigella* was significantly negatively correlated with BarH-like homeobox 2 (BARX2), Kcnma calcium-activated potassium channel subunit alpha-1 (KCNMA1), ACTN2, and PHGDH. In addition, *Subdoligranulum*, *Corynebacterium_1*, and *Terrisporobacter* had no significant correlation with all the differential genes.

## 3. Discussion

The frequent occurrence of DON in cereal feed ingredients has attracted worldwide attention. Previous study reported that ingesting DON could affect intestinal health and nutrient absorption [17]. The intestinal tract is the largest immune organ of animal body, which has many biological functions such as digestion and absorption, detoxification, and immune enhancement. The immunity, the change of diet composition, and intestinal microbiota will directly affect the normal operation of intestinal function. In the present study, a total of 86 DEGs were found in CON vs. DON and DON vs. GPD groups. Among them, 31 DEGs were up-regulated in CON vs. DON group but down-regulated in DON vs. GPD group, and 51 DEGs were down-regulated in CON vs. DON group but up-regulated in DON vs. GPD group, suggesting that these genes may play a key role in alleviating intestinal injury after GAP supplementation. Parts of the DEGs were further confirmed by qRT-PCR, indicating the effectiveness of the screened DEGs. Furthermore, the GO enrichment analysis showed that most of the DEGs were related to metabolic processes, nutritional transportation, transporter activity, and molecular function regulator. PPAR signaling pathway is mainly related to lipid metabolism, adipocyte differentiation, fatty acid metabolism, gluconeogenesis, and body thermogenesis. According to the KEGG pathway enrichment analysis, we found that PPAR signaling pathways, protein digestion and absorption, vitamin digestion and absorption, linoleic acid metabolism, and fat digestion and absorption were significantly influenced by GAP addition, indicating that GAP supplementation could promote the digestion and absorption of nutrients such as protein, fat, and vitamins in the intestine. In these pathways, the DEGs such as HSP90AA1 and CCL16 were up-regulated in CON vs. DON group but down-regulated in DON vs. GPD group. HSP90AA1 is a stress response protein that maintains cellular homeostasis [18]. Seibert et al. [19] found that the expressions of HSP90AA1 and HSPA1A in ovaries of sows were significantly increased after both heat stress and LPS challenge, indicating that the effect of heat stress on ovaries was partially mediated by LPS. CCL16 is a strong pro-inflammatory chemokine, which has chemotactic effect on monocytes and lymphocytes [20]. The changes of these genes indicated that DON exposure induced stress response and inflammation, thus leading to intestinal disorders, while GAP supplementation alleviated intestinal inflammation and disorders. On the other hand, the DEGs including PLIN1, PLIN4, ADIPOQ, FABP4, PHGDH, and FUT2 were down-regulated in CON vs. DON group but up-regulated in DON vs. GPD group. PLIN1 and PLIN4 are the member of perilipin-family proteins related to lipid droplet (LD) surface proteins, which play vital role during fat metabolism of adipose tissue lipolysis and fat storage in adipocytes [21,22]. Fatty acid binding protein 4 (FABP4), a subtype of fatty acid-binding protein family, is a key transmitter of lipid metabolism and inflammatory reaction [23]. ADIPOQ, also known as adiponectin and mainly expressed in adipose tissue, is associated with lipid metabolism and hormone production, including fatty acid uptake, binding, transport, oxidation, and lipoprotein assembly [24]. Furthermore, PPI analysis revealed that ADIPOQ was the hub gene that had the most interaction with many genes in the CON vs. DON group. The down-regulated genes indicated that DON exposure inhibited lipid metabolism, fatty acid transport, and oxidation in the gut of piglets, whereas GAP addition promotes lipid metabolism. In addition, the phosphoglycerate dehydrogenase (PHGDH) is the first branching enzyme in the glycolytic and serine biosynthesis pathway [25]. FUT2 is fucosyltransferase 2, and the intestinal mucosal fucosylation can induce or alleviate intestinal inflammation by affecting the body’s signaling pathway and immune system [26]. Pham et al. [27] found that the counts of *Escherichia coli* and *Clostridium bacillus* in the feces of FUT2-deficient mice were significantly increased, while the counts of Bifidobacterium and *Lactobacillus* were significantly decreased. In the present study, GAP addition significantly increased the expressions of PHGDH and FUT2. It can be concluded that DON exposure induced intestinal inflammation and inhibited lipid metabolism and serine synthesis, while GAP addition could effectively alleviate intestinal inflammation, promote lipid metabolism and glycolysis, and enhance immunity.

Intestinal microbiota is the basis of animal digestion, absorption, and metabolism, and its balance is considered as a key factor for intestinal health, while microbiota dysbiosis is an important factor affecting intestinal gene expression. Previous study showed that DON could impact the composition and diversity of intestinal microorganisms in piglets [28]. However, the probiotics and some plant extracts addition could promote intestinal development and colonization of beneficial bacteria and regulate the composition of intestinal microflora [29,30]. In the current study, we found that DON exposure significantly affected the composition and function of gut microbiota, and dietary GAP supplementation could improve the diversity of gut microbiota. *Firmicutes* and *Proteobacteria* were the two predominant phyla in this study, which was in accordance with the previous results [31]. Many bacteria belonging to *Firmicutes* have been shown to be involved in energy metabolism and the maintenance of intestinal health [32]. The proliferation of *Proteobacteria* is also thought to be a microbiological characteristic of intestinal epithelial dysfunction [33]. The decrease of *Firmicutes* and the increase of *Proteobacteria* in DON group indicated that DON exposure induced intestinal functional impairment, thereby affecting intestinal health. In addition, we found that GAP addition significantly increased *Lactobacillus* abundance, suggesting that GAP could promote colonization of *Lactobacillus* and enhance its ability of adsorption and biodegradation of DON. TNS1 is a focal adhesion molecule that regulates cell adhesion, migration, and proliferation [34]. Many reports showed that TNS1 is associated with growth and meat quality of weaned piglets [35,36]. CDON is a cell surface receptor that belongs to a subgroup of the immunoglobulin superfamily of cell adhesion molecules [37]. The correlation analysis showed that *Lactobacillus* was positively correlated with TNS1 and CDON, further indicating that *Lactobacillus* is closely related to the immunity, growth, and development of piglets. Moreover, the LefSe analysis showed that many species of uncultured bacteria were enriched in DON-challenged piglets, further indicating that DON could significantly alter microbiota composition. In addition, predictive functions by PICRUSt analysis showed that DON exposure decreased lipid metabolism and increased immune diseases, but GAP supplementation significantly enhanced the immune system. These results reveal that GAP supplementation could improve intestinal microbial communities and enhance immune through promoting colonization of beneficial bacteria during DON exposure.

On the other hand, there is a significant positive correlation between *Rothia* and FUT2, which may be because *Rothia* can metabolize the fucose group of intestinal mucin side chain as a carbon source to adapt to the intestinal environment, thus promoting its proliferation. In addition, we also found that *Escherichia-Shigella* was negatively correlated with BARX2, KCNMA1, ACTN2, and PHGDH. BARX2 is a homologous heteromorphic box transcription factor, which can regulate facial development, gland, chondrogenesis, and neural development [38,39]. It was inferred that the increase of *Escherichia-Shigella* may affect the development of cartilage and nervous system as well as the proliferation of intestinal epithelial cells in weaned piglets. These results indicated that the intestinal gene expression in DON-contaminated diet regulated by GAP supplementation may be associated with the changes in the gut microbiota composition, thus affecting intestinal health.

## 4. Conclusions

The present study demonstrated that dietary exposure to DON altered the intestinal gene expressions related to nutrient metabolism and induced disturbances of intestinal microbiota, while supplementing GAP to DON-contaminated diets in piglets could improve intestinal health and immune system.

## 5. Materials and Methods

### 5.1. Glycyrrhizic Acid and Compound Probiotics Preparation

Glycyrrhizic acid was provided by Henan Delin Biological Products Co. Ltd., Xinxiang, China. *Saccharomyces cerevisiae* (*S. cerevisiae*, CGMCC 2.1542) and *Enterococcus faecalis* (*E. faecalis*, CGMCC1.2135) used in this experiment were purchased from China General Microbiological Culture Collection Center (CGMCC), Beijing, China. They were incubated according to the published protocols [40] and then freeze dried. Based on our preliminary experimental results obtained with orthogonal design in vitro, the optimal ratio of GA and compound probiotics (GAP) were 400 mg/kg GA, 1 × 10^6^ CFU/g *S. cerevisiae* and 1 × 10^6^ CFU/g *E. faecalis.*

### 5.2. Animals and Managements

A total of 120 42-day-old weaned piglets (Landrace × Large White) were randomly divided into three experimental groups with four replicates per group, with ten piglets per replicate (half male and half female). The control group (CON): the piglets fed with a basal diet; the DON-contaminated group (DON): the piglets fed with a DON-contaminated diet (1040 µg/kg DON); the GAP+DON group (GPD): the piglets fed with a DON-contaminated diet (1040 µg/kg DON) supplemented with 400 mg/kg GA, 1 × 10^6^ CFU/g *S. cerevisiae*, and 1 × 10^6^ CFU/g *E. faecalis*. The basal diets were prepared according to the recommended nutrient requirement standard (NRC, 2012). DON-contaminated diets were prepared with the DON-contaminated wheat (2600 μg/kg). The 40% normal wheat was used to prepare the basal diet, and the same amount of moldy wheat was used to replace the normal wheat in the basal diet for preparing the moldy diet with 1040 µg/kg DON. All piglets were raised individually in environment-controlled cages. Diets and water were given ad libitum during the whole experimental period of 28 days. All animal experimental procedures and animal welfare policies were approved by the Animal Care and Use Committee of Henan Agricultural University (SKLAB-B-2010-003-01; approval date: 10 March 2010).

At the end of the trial, three boars in CON, DON, and GPD groups with similar growth conditions were slaughtered. After slaughter, all organs were carefully removed, washed with normal saline, dried with absorbent paper, and weighed to calculate the organ index. About 2 g jejunum and some uncontaminated intestinal contents were snap-frozen immediately in liquid nitrogen and stored at −80 °C for further analysis.

### 5.3. RNA-Seq Analysis

Total RNA was isolated using Trizol (Invitrogen, NY, USA) according to the manufacturer’s instructions. The RNA quantity, purity, and integrity were detected by the Agilent 2100 Bioanalyzer (Agilent, CA, USA) and agarose gel electrophoresis, respectively. The RNA-seq procedures including library construction and sequencing were identical to our previous study [41]. In brief, mRNA was enriched, fragmented, and transcripted into cDNA. Then, the synthesized cDNA was purified by the PCR purification kit (Qiagen, Venlo, The Netherlands), followed by end repair, poly (A) addition, and sequencing adapter ligation. Finally, cDNA libraries were sequenced on the Illumina HiSeq 4000 platform (Illumina, CA, USA) with a paired-end 150 bp sequencing strategy. Sample clean reads were aligned to the reference porcine genome assembly Sus scrofa 11.1 using HISAT2 [42]. Read numbers for each gene were calculated using HTSeq with union strategy [43].

The gene expression levels were estimated by calculating the value of fragments per kilobase of exon per million reads mapped (FPKM). DEGs were judged with the absolute value of log2 (fold change) >1 and the threshold of *p*-value < 0.05. Then, GO annotation, KEGG pathway enrichment and PPI network analyses were performed by using the GOseq R-package, KOBAS software, and the Search Tool for the Retrieval of Interacting Genes/Proteins database (STRING, version 11.0, https://string-db.org, accessed on 13 March 2021), respectively.

### 5.4. QRT-PCR Validation

Total RNA (1 mg) from jejunum sample was converted into cDNA for RT-PCR using StarScript II First-strand cDNA Synthesis Mix (TaKaRa, Dalin, China). The RT-PCR amplification was performed using the SYBR Green PCR Master Mix (Takara, Dalian, China) by a CFX Connect™ Real-Time PCR Detection System (BioRad, Hercules, CA, USA). The primer sequences of eight genes are shown in Table 1. The relative gene expression levels were normalized with GAPDH using the 2^−ΔΔCt^ method [44].

### 5.5. 16S rRNA Gene Sequencing Analysis

The DNA from 300 mg jejunal contents of each sample was isolated using Soil DNA Kit (Omega Biotek, Norcross, GA, USA) according to the manufacturer’s instructions. The V3-V4 region of the 16S rRNA gene was amplified by polymerase chain reaction (PCR) with universal primers 341F 5′-CCTACGGGRSGCAGCAG-3′ and 806R 5′-GGACTACVVGGGTAT CTAATC-3′ using KAPA HiFi Hotstart ReadyMix PCR kit containing 10 ng of template DNA. The qualified PCR products were quantified by Qubit and then pooled in equal concentration to construct the sequencing library. The sequencing library was then sequenced on the Illumina Hiseq 2500 platform with a paired-end 250 (PE250) strategy according to the manufacturer’s recommendations.

The raw fastq reads were merged by FLASH (v1.2.11) [45] with a minimal overlap of 10 bp and other default parameters. Then, the merged sequences were quality-filtered by Trimmomatic (v0.33) [46], and the chimeras were removed by UCHIME (v8.1) [47] with default settings. The clean sequences were assigned to OTUs at a cut-off of 97% sequence similarity with USEARCH (v7.0.1090) [48]. Every representative sequence, the highest abundance sequence in each OTU was assigned taxonomies by RDP Classifier at an 80% confidence threshold [49]. Subsequent bioinformatics analyses were conducted with the QIIME software package (v1.9.1) [50]. Alpha diversity was evaluated by ACE, Chao 1 (species richness), and Simpson and Shannon (diversity) indices.

### 5.6. Statistical Analysis

All data were expressed as mean ± standard deviation. Statistical analysis was performed by univariate (ANOVA) with Tukey’s posttest using SPSS 20.0 software. Multiple comparisons were performed using Duncan test. Differential OTU abundance analyses at the genus level were compared between two groups and multiple groups using Kruskal–Wallis test. Differences were considered significantly at *p* < 0.05.

## Figures and Tables

**Table 1:** Primer sequences of eight genes for RT-PCR.

Gene	Accession Number	Primer Sequence (5′-3′)
HSP90AA1	NM_213973.2	F: ATCGCCCAGTTGATGTCGTT
		R: TATCGTGAGGGTCCGGTCTT
ANGPTL4	NM_001038644.1	F: TGTTTGAAGAGGGAGAGCGG
		R: TGGTTAAAGTCCACCGAGCC
CHORDC1	NM_001113446.1	F: GCCACCAGAACCAGTCAAAC
		R: TGTCATTGGTTCATCTGGGCT
HSPH1	NM_001097504.1	F: TGAAAGTCAAGGTGCGAGTCA
		R: GCTTCACTGTTGTCTTGCTGG
FUT2	NM_214069.1	F: TAAGCACTGATGTCGGCTGG
		R: CTCCCTGTGCCTTGGAAGTG
PHDGDH	NM_001123162.1	F: GTCCTACCAGACCTCAGTGG
		R: ATGGAACTGGAAAGCCTCAGT
FABP4	NM_001002817.1	F: ACGGCTTCTTTCTCACCTTGA
		R: AGCCCACTCCCACTTCTTTC
ADIPOQ	NM_214370.1	F: GTTGAAGGTCCCCGAGGTTT
		R: CCACACTGAATGCTGAACGG
GAPDH	NM_001206359.1	F: ATGACCACAGTCCATGCCATC
		R: CCTGCTTCACCACCTTCTTG

## Data Availability

Data sharing not applicable.

## Supplementary Materials

The following supporting information can be downloaded at: https://www.mdpi.com/article/10.3390/toxins14120856/s1, Table S1: The summary of 86 co-expressed DEGs in CON vs. DON and DON vs. GPD.
