# Supplementary material for: Glycyrrhizic Acid and Compound Probiotics Supplementation Alters the Intestinal Transcriptome and Microbiome of Weaned Piglets Exposed to Deoxynivalenol
Source: Toxins (Basel). 2022 Dec 4;14(12):856. doi: 10.3390/toxins14120856 (PMC9783239; doi:10.3390/toxins14120856)
Supplement: Supplementary file 1 [file toxins-14-00856-s001.zip › toxins-2038624-supplementary.pdf]

toxins

MDPI

Supplementary Material: Glycyrrhizic Acid and Compound Probiotics Supplementation Alters the Intestinal Transcriptome and Microbiome of Weaned Piglets Exposed to Deoxynivalenol

Xiaoxiang Xu, Juan Chang, Ping Wang, Chaoqi Liu, Mengjie Liu, Ting Zhou, Qingqiang Yin and Guorong Yan

Table S1. The summary of 86 co-expressed DEGs in CON *vs*. DON and DON vs. GPD.

Gene_ID	CON (FPKM)	DON (FPKM)	GPD (FPKM)	CON vs. DON	DON vs. GPD	Gene Name
ENSSSCG00000029879	0.33	1.42	15.69	up	up	LTF
ENSSSCG00000001724	0.68	9.13	4.06	up	down	—
ENSSSCG00000002535	333.49	816.31	370.53	up	down	HSP90AA1
ENSSSCG00000003970	25.40	57.35	24.29	up	down	GUCA2B
ENSSSCG00000004584	7.61	28.22	14.57	up	down	GCNT3
ENSSSCG00000007554	10.99	32.75	4.73	up	down	ZFAND2A
ENSSSCG00000009334	41.12	158.48	32.43	up	down	HSPH1
ENSSSCG00000009673	0.81	3.08	1.13	up	down	NUGGC
ENSSSCG00000010432	0.17	4.06	1.96	up	down	ASAH2
ENSSSCG00000010437	1.02	17.65	7.10	up	down	PAPSS2
ENSSSCG00000010686	9.85	31.07	7.33	up	down	BAG3
ENSSSCG00000013102	0.13	0.97	0.27	up	down	—
ENSSSCG00000013530	1.09	19.20	8.06	up	down	—
ENSSSCG00000013599	3.57	9.84	5.07	up	down	ANGPTL4
ENSSSCG00000014934	10.23	25.78	10.48	up	down	CHORDC1
ENSSSCG00000015895	0.18	5.30	1.61	up	down	GCG
ENSSSCG00000016609	0.00	3.00	0.25	up	down	SLC13A1
ENSSSCG00000017163	0.57	11.84	5.98	up	down	ENPP7
ENSSSCG00000017798	0.26	2.84	0.39	up	down	TMIGD1
ENSSSCG00000020872	21.48	47.34	17.46	up	down	—
ENSSSCG00000025021	8.41	22.18	10.54	up	down	—
ENSSSCG00000025992	6.32	20.73	8.47	up	down	ENPP3
ENSSSCG00000026594	0.00	13.13	0.57	up	down	—
ENSSSCG00000029160	213.44	772.76	113.00	up	down	—
ENSSSCG00000031106	1.52	5.29	2.46	up	down	PLA2G2D
ENSSSCG00000033146	0.77	3.52	1.15	up	down	CD163
ENSSSCG00000034167	0.42	1.14	0.42	up	down	SLC5A3
ENSSSCG00000036825	2.43	11.01	3.26	up	down	—
ENSSSCG00000037358	5.47	47.80	161.54	up	up	HPS5
ENSSSCG00000040940	0.00	6.31	0.00	up	down	CCL16
Sus_scrofa_newGene_384	0.07	5.30	0.33	up	down	—
Sus_scrofa_newGene_4934	1.26	2.59	0.96	up	down	—
Sus_scrofa_newGene_5253	0.07	1.30	0.20	up	down	—
ENSSSCG00000001068	3.12	1.14	3.11	down	up	CAP2
ENSSSCG00000001570	3.07	0.79	2.29	down	up	PI16
ENSSSCG00000001844	1.43	0.36	1.26	down	up	PLIN1

ENSSSCG00000003048	2.28	0.33	1.75	down	up	—
ENSSSCG00000003145	21.53	4.71	15.53	down	up	FUT2
ENSSSCG00000004336	1.07	0.39	1.07	down	up	EPHA7
ENSSSCG00000004366	0.95	0.29	0.90	down	up	BVES
ENSSSCG00000004520	0.82	0.31	0.95	down	up	MAPK4
ENSSSCG00000004630	0.70	0.20	0.66	down	up	SCG3
ENSSSCG00000006140	49.13	8.28	18.87	down	up	CA2
ENSSSCG00000006141	2.99	0.29	1.65	down	up	CA3
ENSSSCG00000006477	0.26	0.06	0.33	down	up	BCAN
ENSSSCG00000006717	10.33	4.78	21.73	down	up	PHGDH
ENSSSCG00000006748	1.34	0.58	1.61	down	up	TSPAN2
ENSSSCG00000007058	1.80	0.78	2.04	down	up	PLCB4
ENSSSCG00000007436	2.81	0.43	2.12	down	up	MMP9
ENSSSCG00000009004	1.60	0.23	1.23	down	up	SFRP2
ENSSSCG00000009111	8.47	4.09	10.29	down	up	SYNPO2
ENSSSCG00000010144	2.35	0.88	3.62	down	up	ACTN2
ENSSSCG00000010325	1.78	0.74	1.87	down	up	KCNMA1
ENSSSCG00000010451	10.40	5.03	16.00	down	up	IFIT2
ENSSSCG00000011640	3.01	0.21	1.11	down	up	TF
ENSSSCG00000011813	3.41	0.84	2.03	down	up	P3H2
ENSSSCG00000013512	1.34	0.57	1.37	down	up	PLIN4
ENSSSCG00000014117	2.12	0.59	1.92	down	up	THBS4
ENSSSCG00000015069	149.06	65.92	36.50	down	down	—
ENSSSCG00000016823	2.41	0.68	2.05	down	up	—
ENSSSCG00000017311	1.60	0.38	1.71	down	up	MAPT
ENSSSCG00000022504	0.60	0.22	0.61	down	up	CDON
ENSSSCG00000023279	1.39	0.48	1.98	down	up	SH3TC2
ENSSSCG00000023934	3.96	1.55	3.50	down	up	KCNIP4
ENSSSCG00000024018	10.34	2.63	6.55	down	up	SLC16A3
ENSSSCG00000027669	8.30	3.39	8.51	down	up	TNS1
ENSSSCG00000028341	0.48	0.13	0.67	down	up	—
ENSSSCG00000030260	6.13	2.74	7.11	down	up	PFN2
ENSSSCG00000030655	1.20	0.52	2.00	down	up	MAMDC2
ENSSSCG00000031037	1053.67	314.74	1029.62	down	up	—
ENSSSCG00000033822	3.48	0.31	2.31	down	up	THRSP
ENSSSCG00000034731	6.01	0.37	7.35	down	up	—
ENSSSCG00000035223	14.78	5.68	19.35	down	up	—
ENSSSCG00000036157	2.58	0.62	2.48	down	up	BARX2
ENSSSCG00000037214	15.85	1.98	12.34	down	up	—
ENSSSCG00000038521	9.43	2.00	5.09	down	up	CHAC1
ENSSSCG00000038606	1.32	0.30	1.52	down	up	—
ENSSSCG00000038969	10.97	5.01	16.33	down	up	DMPK
ENSSSCG00000039103	5.64	0.94	3.20	down	up	ADIPOQ
ENSSSCG00000039339	2.73	0.68	2.06	down	up	—
ENSSSCG00000040681	5.24	1.29	4.15	down	up	FABP4
ENSSSCG00000040689	446.15	194.71	76.76	down	down	APOA4
ENSSSCG00000040735	18.49	7.52	16.72	down	up	DDAH1
ENSSSCG00000042270	7.43	0.45	3.30	down	up	—
Sus_scrofa_newGene_5263	5.97	1.51	6.09	down	up	—
Sus_scrofa_newGene_7452	5.74	1.05	2.69	down	up	—
